# The effect of prime emulsion components as a function of equilibrium headspace concentration of soursop flavor compounds

**DOI:** 10.1186/1752-153X-8-23

**Published:** 2014-04-03

**Authors:** Kok Whye Cheong, Chin Ping Tan, Hamed Mirhosseini, Wai Yee Joanne-Kam, Nazimah Sheikh Abdul Hamid, Azizah Osman, Mahiran Basri

**Affiliations:** 1 Department of Food Technology, Faculty of Food Science and Technology, Universiti Putra Malaysia, 43400, Serdang, Selangor, Malaysia; 2 Department of Pharmaceutical Chemistry, School of Pharmacy, International Medical University, No. 126, Jalan Jalil Perkasa 19, Bukit Jalil, 57000 Kuala Lumpur, Malaysia; 3 Flavor Inn Corporation Sdn. Bhd., No. 6, Jalan Anggerik Mokara 31/54, Kota Kemuning, Seksyen 31, 40460 Shah Alam, Selangor, Malaysia; 4 School of Applied Sciences, AUT University, 34 St. Paul Street, Auckland, New Zealand; 5 Department of Food Science, Faculty of Food Science and Technology, Universiti Putra Malaysia, 43400 Serdang, Selangor, Malaysia; 6 Department of Chemistry, Faculty of Science, Universiti Putra Malaysia, 43400 Serdang, Selangor, Malaysia

**Keywords:** Mixture design, Modified starch, Whey protein isolate, Soursop beverage emulsion, Polysaccharide-protein interactions

## Abstract

**Background:**

Perceptions of food products start when flavor compounds are released from foods, transported and appropriate senses in the oral and nose are triggered. However, the long-term stability of flavor compounds in food product has been a major concern in the food industry due to the complex interactions between key food ingredients (*e.g.*, polysaccharides and proteins). Hence, this study was conducted to formulate emulsion-based beverage using natural food emulsifiers and to understand the interactions between emulsion compositions and flavor compounds.

**Results:**

The influences of modified starch (*x*_*1*_), whey protein isolate (*x*_*2*_), soursop flavor oil (*x*_*3*_) and deionized water (*x*_*4*_) on the equilibrium headspace concentration of soursop volatile flavor compounds were evaluated using a four-component with constrained extreme vertices mixture design. The results indicated that the equilibrium headspace concentration of soursop flavor compounds were significantly (*p* < 0.05) influenced by the matrix and structural compositions of the beverage emulsions. Interface formed using modified starch and whey protein isolate (WPI) proved to be capable of inhibiting the release of volatile flavor compounds from the oil to the aqueous phase. Modified starch could retard the overall flavor release through its hydrophobic interactions with volatile flavor compounds and viscosity enhancement effect. Excessive amount of modified starch was also shown to be detrimental to the stability of emulsion system. However, both modified starch and WPI showed to be a much more effective barrier in inhibiting the flavor release of flavor compounds when used as individual emulsifier than as a mixture.

**Conclusions:**

Overall, the mixture design can be practical in elucidating the complex interactions between key food components and volatile flavor compounds in an emulsion system. These studies will be useful for the manufacturers for the formulation of an optimum beverage emulsion with desirable emulsion properties and desirable flavor release profile.

## Background

Flavor is one of the most important components responsible for the overall sensory properties of taste and smell in any food products (*e.g.*, soft drinks). Among the many organoleptic quality components, such as color, rheological properties or packaging, flavor takes a particular place through stimulating the odor and taste receptors when eating [1]. Therefore, flavor plays an important role in consumer satisfaction, which will subsequently drive consumers’ acceptance and influences the continued consumption of beverages [2]. However, due to the volatility and delicate properties of volatile flavor compounds, encapsulation of flavor compounds prior to its final application as food ingredients is often done [3].

A food emulsion may be defined as a heterogeneous system of two immiscible liquid phases, where one of the liquids being dispersed into the other phase [4]. Because flavor can be one of the most expensive and delicate ingredients in beverage formulations, protection of these labile compounds from degradation, oxidation and evaporation has often a top priority for manufacturers, while allowing a controlled release from the food matrix [3]. In the fruit-flavored beverage manufacturing, emulsions have often been used as a medium to carry oil-based flavoring compounds (*e.g.*, beverage emulsion) or to impart turbidity (*e.g.*, cloud emulsion) to the final products [2]. Various properties of the flavor compounds, such as molecular size, functional groups, shape and volatility, will determine its interactions with food components and, hence its retention in the food matrix [5]. Beverage emulsions are also unique as they are normally prepared in a concentrated form and then diluted with sugar solution to yield finished beverage products [6]. This unique class of emulsion must have a high degree of stability in both the concentrated and diluted form, given that as little as 20 mg/L dispersed oil phase may be contained in the finished product [7]. Beverage emulsion is a thermodynamically unstable system as the free energy during the formation of dispersions is often positive, thus the system is susceptible to destabilize through various mechanisms such as creaming, flocculation, coalescence, Ostwald ripening and sedimentations [4]. As the surface contact between oil and water is energetically unfavorable, kinetically stable emulsions can be produced by the addition of emulsifiers, including synthetic surfactants, proteins, polysaccharides or phospholipids. Emulsifiers can be used to overcome the activation energy of the system by reducing the interfacial tension between the two layers and thus, enhancing its stability over a longer period of time [8].

Gum arabic is the most commonly used emulsifier for the stabilization of beverage emulsions in the soft drink industry but problems associated with the variations in market price, reliable sources and consistent quality of gum arabic have led many scientists to search of a replacement for use in flavored beverages [6]. It has been suggested that new sources of natural biopolymer such as alginate, mannan, corn fiber gum, durian seed gum, fish protein hydrolysate and buckwheat protein can be used as alternative emulsifiers [9-14]. Nevertheless, among the numerous food biopolymers, hydrophobically modified starch has been touted as one of the most promising biopolymer replacement for gum arabic [15]. Previous studies have indicated that modified starch is mildly anionic in aqueous solutions and has a surface activity that is almost as high as that of gum arabic [16]. The hydrophobic octenyl succinic anhydride (OSA) anchor itself to the oil–water (o/w) interface, while hydrophilic starch chains extend into the aqueous solution to prevent droplets coalescence and flocculation through steric repulsion mechanism [17]. They formed a strong film at the o/w interface, capable of resisting re-agglomeration of newly formed emulsion droplets [18]. One important aspect of modified starch is its ability to act as both an emulsifier and a thickener in stabilizing the flavor emulsion system [19].

Proteins have often been applied as emulsifiers in food emulsions because they naturally have hydrophobic and hydrophilic regions and are therefore surface-active [20]. The ability to scavenge free radicals to inhibit lipid oxidation through cysteine residues, disulfide bonds and thiol function groups has also spurred interest in formulating whey protein isolate (WPI)-stabilized emulsion systems [8]. Consequently, WPI have found much favor in the food emulsion industry due to their unique properties, such as antioxidant activity and high nutritional value, which can improve health and prevent disease [8,21]. Whey protein isolate is able to adsorb rapidly onto the o/w interface to form a protective film to provide protection for emulsion droplets through electrostatic repulsion [4]. Additionally, WPI-stabilized emulsion containing citral, a major flavor component of citrus oils, was reported to be much more stable against oxidation than comparable emulsion produced with gum arabic [22].

Flavor release is defined as a flavor compound transport process from the matrix to the vapor phase [23]. Thus, a good knowledge of the physicochemical interactions occurring between flavor compounds and other major food components is required for the control of food flavoring and, more particularly, for understanding the phenomena involved in the release of aroma compounds in the mouth. In addition, the composition of the food matrix will determine the extent and type of flavor compounds it is inclined to bind [5]. The variations of food components in different food matrices have contribute significantly to different interactions between the flavor compounds with other food components, which consequently influence the equilibrium headspace concentration of flavor compounds [1]. Interactions between flavor compounds and other major food components such as proteins, lipids and polysaccharides, have also been widely documented [24-26]. Mirhosseini et al. [26] reported that gum arabic and xanthan gum had a significant (*p* < 0.05) effect on the total flavor release and release pattern of α-pinene and octanal, though the different degree of interactions were dependent on the physicochemical properties of the flavor compounds [27]. While Mao et al. [24] and Chen [28] reported on the influence of fat content on the emulsion texture, which consequently affects the creaminess, smoothness and how flavor is perceived during consumption. The presence of proteins in the system may also often decrease the volatility of flavor compounds through reversible and irreversible binding mechanisms [29]. In addition, the stability of flavor compounds has often been associated with the quality and acceptability of food products [3].

The stability of flavor compounds and its release pattern from food matrix (*e.g.*, emulsions) have been extensively studied due to its impact on the quality and acceptability of food products [30-32]. Nevertheless, there is a relatively poor understanding of how different composition of major food components such as starch and protein will affect the physicochemical properties and equilibrium headspace concentration of soursop flavor compounds in a beverage emulsion system. In addition, the lack of systematic studies of these interactions has also prompted a need for this study. Therefore, a four-component [modified starch (5-12% w/w), WPI (0-2% w/w), soursop oil content (5-15% w/w) and deionized water (67.4-86.4% w/w)] with constrained extreme vertices design was used for a systematic and organized study on the effect of emulsion components on the equilibrium headspace concentration of soursop volatile flavor compounds. The information and knowledge gained from this study will contribute positively for the beverage industries.

## Results and discussion

### Preliminary study

Based on previous study [33], a total of 37 volatile compounds were identified by GC × GC-TOFMS as key volatile compounds of soursop fruit. However, in this study, a synthetic oil-based soursop flavor was used as the oil phase for the formation of oil-in-water emulsion system. This would facilitate the studies of the efficiencies of both modified starch and WPI as emulsifiers and as protective films to impede the transfer of volatile soursop flavor compounds into vapor phase. Thirteen volatile flavor compounds namely, methyl butanoate, ethyl butanoate, methyl 2-butenoate, 1-butanol, methyl hexanoate, (*E*)-2-hexenal, ethyl hexanoate, methyl 2-hexenoate, (*Z*)-3-hexen-1-ol, linalool, butanoic acid, hexanoic acid and methyl (*E*)-cinnamate which represented about 85% of the total flavor compounds of soursop fruit were chosen and blended to produce the synthetic soursop flavor. Thus, these flavor compounds will be used as the main representative of the soursop volatile flavor compounds and the peak area of these flavor compounds were considered as response variables.

Initial experiments were also conducted to study the influence of several variables and to establish the most favorable emulsion preparation conditions (*e.g.*, speed of the homogenizer, duration of the shearing and pressure of the high-pressure homogenizer). The results showed that a fine emulsification with a good creaming stability could be achieved by mixing the emulsion for 1 min using a high-shear homogenizer (6,000 rpm) before being sent through a high-pressure homogenizer for 2 cycles at 200 bar.

### Fitting the regression models

The use of the mixture design (Table 1) has allowed the study of the possible interaction effects between main emulsion components as a function of the equilibrium headspace concentration of soursop flavor compounds. The estimated regression coefficients of four dependent variables (modified starch, WPI, soursop oil and deionized water), along with the corresponding *R*^*2*^, adjusted *R*^*2*^ and *p*-value of regressions are given in Table 2. The individual significance probabilities of each parameter term and the *F*-ratio are shown in Table 3. Each response (*Y*_i_) was assessed as a function of interaction effects of modified starch (*x*_1_), WPI (*x*_2_), soursop flavor oil (*x*_3_) and deionized water (*x*_4_).

**Table 1:** Four components constrained mixture design

**Run**	**Modified starch (% w/w)**	**Whey protein isolate (% w/w)**	**Flavor oil (% w/w)**	**Water (% w/w)**
1	5.00	2.00	15.00	74.40
2	10.25	1.50	7.50	77.15
3	10.25	0.50	7.50	78.15
4	5.00	2.00	15.00	74.40
5	5.00	2.00	5.00	84.40
6	5.00	0.00	5.00	86.40
7	10.25	0.50	12.50	73.15
8	5.00	0.00	5.00	86.40
9	12.00	2.00	5.00	77.40
10	12.00	2.00	15.00	67.40
11	6.75	1.50	7.50	80.65
12	6.75	1.50	7.50	80.65
13	10.25	0.50	12.50	73.15
14	10.25	0.50	7.50	78.15
15	12.00	0.00	5.00	79.40
16	6.75	0.50	12.50	76.65
17	12.00	2.00	15.00	67.40
18	5.00	2.00	5.00	84.40
19	5.00	0.00	15.00	76.40
20*	8.50	1.00	10.00	76.90
21	6.75	0.50	7.50	81.65
22	12.00	0.00	5.00	79.40
23	6.75	0.50	12.50	76.65
24	10.25	1.50	12.50	72.15
25	10.25	1.50	7.50	77.15
26	6.75	1.50	12.50	75.65
27	12.00	2.00	5.00	77.40
28	10.25	1.50	12.50	72.15
29	6.75	1.50	12.50	75.65
30	12.00	0.00	15.00	69.40
31	5.00	0.00	15.00	76.40
32	6.75	0.50	7.50	81.65
33*	8.50	1.00	10.00	76.90
34	12.00	0.00	15.00	69.40

*center point.

**Table 2:** **Regression coefficients, *R***^***2***^**, adjusted *R***^***2 ***^**and probability values for the final reduced models**

**Regression coefficients**	**Methyl butanoate (*Y***_**1**_**)**	**Ethyl butanoate (*Y***_**2**_**)**	**Methyl 2-butenoate (*Y***_**3**_**)**	**1-Butanol (*Y***_**4**_**)**	**Methyl hexanoate (*Y***_**5**_**)**	**( *E *)-2-Hexenal (*Y***_**6**_**)**	**Ethyl hexanoate (*Y***_**7**_**)**	**Methyl 2-hexenoate (*Y***_**8**_**)**	**( *Z *)-3- Hexen-1-ol (*Y***_**9**_**)**	**Linalool (*Y***_**10**_**)**	**Butanoic acid (*Y***_**11**_**)**	**Hexanoic acid (*Y***_**12**_**)**	**Methyl ( *E *)-cinnamate (*Y***_**13**_**)**
*b*_1_	684	-308	-14971943	-23	-22139939	-4518678	-832147	-123565007	-2306039	-2652877	-21532	-20272	-132872
*b*_2_	-94586	-7369	-1456816	21661	-1171614	-471769	-100453	-10924092	-253322	-303103	-4913	-1731	-17110
*b*_3_	5605	570	5306117	340	3901129	1577380	317050	39123203	802492	922911	870	705	44197
*b*_4_	341	54	-48735	4	-106497	-15210	-2572	-447916	-7805	-9112	-176	-177	-503
*b*_12_	-407882	-47168	-2173868	-4088	-319723	-646450	-136281	-14506798	-324820	-397701	-20804	-18969	-21533
*b*_13_	3098	-	-534996	-	-942085	-153867	-27538	-4596158	-79378	-79924	1105	835	-2589
*b*_14_	-	-	134465	-	259193	40684	7092	1181480	20827	23575	282	264	1163
*b*_23_	228757	29506	2148382	1273	1437389	636873	128996	15712955	325659	373674	10777	10984	17876
*b*_24_	-	-	-	-241	-	-	-	-	-	-	-	-	-
*b*_34_	-	-	-	-4	-	-	-	-	-	-	-	-	-
*b*_*1*23_	2288	238	-	32	-	-	-	-	-	-	133	123	-
*b*_*1*24_	5274	610	-	49	-	-	-	-	-	-	271	245	-
*b*_*1*34_	-49	-	-	-	-	-	-	-	-	-	-14	-10	-
*b*_234_	-3074	-398	-	-21	-	-	-	-	-	-	-146	-151	-
*b*_*1123*_	-	-	-	-	-	-	-	-	-	38	-	-	7
*b*_*1124*_	-	-	-312	-	-360	-100	-21	-2508	-54	-60	-	-	-3
*b*_*1134*_	-	-	1308	-	1124	388	77	9837	198	225	-	-	11
*b*_*2234*_	-	-	-14831	-	-9959	-4401	-890	-108565	-2248	-2560	-	-	-120
*b*_*1223*_	-	-	5845	-	3551	1795	342	42061	871	731	-	-	-
*b*_*1224*_	-	-	17980	-	6977	5413	1128	125490	2757	3306	-	-	174
*b*_*1334*_	-	-	-577	-	-	-174	-38	-3757	-88	-109	-	-	-6
Model	Special cubic	Special cubic	Special quartic	Special cubic	Special quartic	Special quartic	Special quartic	Special quartic	Special quartic	Special quartic	Special cubic	Special cubic	Special quartic
*R*^2^	0.981	0.898	0.957	0.943	0.982	0.990	0.831	0.952	0.978	0.937	0.978	0.940	0.866
*R*^2^ (adj.)	0.970	0.860	0.924	0.915	0.970	0.982	0.709	0.916	0.960	0.885	0.964	0.906	0.769
Regression (*p*-value)	0.000^a^	0.000^a^	0.000^a^	0.000^a^	0.000^a^	0.000^a^	0.000^a^	0.000^a^	0.000^a^	0.000^a^	0.000^a^	0.000^a^	0.000^a^

*b*_i_ represents the estimated regression coefficients for the main linear effects. *b*_ij_, *b*_ijk_ and *b*_ijkl_ represents the estimated regression coefficient for the interaction effects, respectively.

1: Modified starch; 2: WPI; 3: Soursop oil; 4: Deionized water.

^a^Significant (*p* < 0.05).

**Table 3:** **The significance probability ( *p *-value, *F*-value) of regression coefficients in the final reduced models**

**Variables (response area, cm**^**2**^**)**	**Interaction effects**																	
		***x***_**1**_ ***x***_**2**_	***x***_**1**_ ***x***_**3**_	***x***_**1**_ ***x***_**4**_	***x***_**2**_ ***x***_**3**_	***x***_**2**_ ***x***_**4**_	***x***_**3**_ ***x***_**4**_	***x***_**1**_ ***x***_**2**_ ***x***_**3**_	***x***_**1**_ ***x***_**2**_ ***x***_**4**_	***x***_**1**_ ***x***_**3**_ ***x***_**4**_	***x***_**2**_ ***x***_**3**_ ***x***_**4**_	***x***_**1**_ ***x***_**1**_ ***x***_**2**_ ***x***_**3**_	***x***_**1**_ ***x***_**1**_ ***x***_**2**_ ***x***_**4**_	***x***_**1**_ ***x***_**1**_ ***x***_**3**_ ***x***_**4**_	***x***_**2**_ ***x***_**2**_ ***x***_**3**_ ***x***_**4**_	***x***_**1**_ ***x***_**2**_ ***x***_**2**_ ***x***_**3**_	***x***_**1**_ ***x***_**2**_ ***x***_**2**_ ***x***_**4**_	***x***_**1**_ ***x***_**3**_ ***x***_**3**_ ***x***_**4**_
Methyl butanoate	*p*-value	0.000*	0.018*	-	0.000*	-	-	0.000*	0.000*	0.023*	0.000*	-	-	-	-	-	-	-
	*F*-value	37.700	6.76		62.410			22.000	37.700	6.150	62.568							
Ethyl butanoate	*p*-value	0.001*	-	-	0.000*	-	-	0.003*	0.001*	-	0.000*	-	-	-	-	-	-	-
	*F*-value	14.364			17.808			11.628	14.364		17.978							
Methyl 2-butenoate	*p*-value	0.006*	0.013*	0.016*	0.007*	-	-	-	-	-	-	-	0.018*	0.008*	0.007*	0.005*	0.007*	0.006*
	*F*-value	10.049	7.784	7.182	9.364								6.684	9.120	9.364	10.240	9.425	9.672
1-Butanol	*p*-value	0.002*	-	-	0.024*	0.038*	0.013*	0.000*	0.002*	-	0.009*	-	-	-	-	-	-	-
	*F*-value	12.816			5.904	4.884	7.453	17.978	12.390		8.410							
Methyl hexanoate	*p*-value	0.000*	0.000*	0.000*	0.000*	-	-	-	-	-	-	-	0.000*	0.000*	0.000*	0.000*	0.000*	-
	*F*-value	53.582	76.738	81.000	74.650								74.132	77.969	74.477	75.516	75.690	
(*E*)-2-Hexenal	*p*-value	0.000*	0.000*	0.000*	0.000*	-	-	-	-	-	-	-	0.000*	0.000*	0.000*	0.000*	0.000*	0.000*
	*F*-value	24.900	23.717	25.100	25.000								23.136	25.301	25.100	29.268	24.900	24.010
Ethyl hexanoate	*p*-value	0.000*	0.003*	0.003*	0.001*	-	-	-	-	-	-	-	0.001*	0.001*	0.001*	0.001*	0.001*	0.000*
	*F*-value	18.063	11.560	11.560	16.484								15.288	15.840	16.403	17.057	17.472	18.490
Methyl 2-hexenoate	*p*-value	0.000*	0.000*	0.000*	0.000*	-	-	-	-	-	-	-	0.000*	0.000*	0.000*	0.000*	0.000*	0.000*
	*F*-value	31.472	39.816	39.816	35.046								32.036	36.361	35.165	37.454	32.490	28.837
(*Z*)-3-Hexen-1-ol	*p*-value	0.000*	0.000*	0.000*	0.000*	-	-	-	-	-	-	-	0.000*	0.000*	0.000*	0.000*	0.000*	0.000*
	*F*-value	28.196	26.214	27.040	28.837								29.268	28.730	28.944	30.470	28.730	27.668
Linalool	*p*-value	0.000*	0.000*	0.000*	0.000*	-	-	-	-	-	-	0.007*	0.000*	0.000*	0.000*	0.000*	0.000*	0.000*
	*F*-value	39.063	25.604	36.603	39.564							9.548	37.088	39.438	39.188	21.160	39.816	37.210
Butanoic acid	*p*-value	0.000*	0.000*	0.000*	0.000*	-	-	0.000*	0.000*	0.000*	0.000*	-	-	-	-	-	-	-
	*F*-value	33.293	68.063	42.120	43.824			26.420	33.989	72.590	44.890							
Hexanoic acid	*p*-value	0.016*	0.005*	0.006*	0.002*	-	-	0.031*	0.016*	0.008*	0.002*	-	-	-	-	-	-	-
	*F*-value	6.970	9.797	9.797	12.390			5.429	7.023	8.762	13.032							
Methyl (*E*)-cinnamate	*p*-value	0.001*	0.000*	0.000*	0.000*	-	-	-	-	-	-	0.000*	0.000*	0.000*	0.000*	-	0.000*	0.001*
	*F*-value	16.403	24.206	49.703	26.010							23.136	29.594	31.923	26.112		19.184	14.213

1: Modified starch; 2: WPI; 3: Soursop oil; 4: Deionized water.

*Significant at *p* < 0.05.

The mixture design analysis has shown that the regression models were significantly (*p* < 0.05) fitted for all of the flavor compounds studied, with relatively high *R*^*2*^ values, ranging between 0.831 to 0.990, which indicated that the regression equations could adequately explain the relationship between the dependent factors and the response variables observed. The results clearly showed that the equilibrium headspace concentration of soursop flavor compounds was significantly (*p* < 0.05) affected by the proportion of main emulsion components. For instance, emulsions containing similar amount of WPI and soursop flavor oil but different amount of modified starch (formulations 28 and 29) showed a significant (*p* < 0.05) different equilibrium headspace concentration of soursop flavor compounds. This could be due to the different interaction effects between flavor compounds and hydrocolloids used in the formulations. In addition, the different degree of interaction is dependent on the physicochemical characteristics of the flavor compounds [34]. Meanwhile, interactions of modified starch and water (*x*_1_ × *x*_4_), and WPI and water (*x*_2_ × *x*_4_) were found to have the most and least significant (*p* < 0.05) effects on the variations of methyl hexanoate and 1-butanol, respectively.

### The interaction effects of bi-components

Interactions between two components such as modified starch and WPI (*x*_1_ × *x*_2_), WPI and water (*x*_2_ × *x*_4_) and soursop oil with water (*x*_3_ × *x*_4_) had significant (*p* < 0.05) effects on the equilibrium headspace concentrations of soursop beverage emulsion (Table 3), whereas the regression coefficients in Table 2 indicated that these interactions would have antagonistic effects on the flavor release from the emulsion matrix. Accordingly, this analysis showed that the films that formed using the combination of both modified starch and WPI (*x*_1_ × *x*_2_) were capable of providing a barrier to inhibit flavor release of volatile flavor compounds. In addition, the negative effect of flavor oil and water (*x*_3_ × *x*_4_) on 1-butanol could be due to the polarity attribute of the compound, which could give it a higher affinity for the water phase than the oil phase.

Meanwhile, the interactions of modified starch and flavor oil (*x*_1_ × *x*_3_) yielded negative (*p* < 0.05) effects on all of the volatile flavor compounds except for methyl butanoate, butanoic acid and hexanoic acid. This result could be explained by the fact that the hydrophobic fraction of the modified starch was able to bind the hydrophobic (log *P* between 1.32 to 3.38) flavor compounds at the o/w interface and, hence, retained them in the oil phase. In addition, the negative effect of the modified starch on the overall flavor release may be attributed to the physical entrapment of the flavor compound molecules within the emulsion matrix through its viscosity enhancement effect, which retards the flavor release of volatile compounds [35,36]. Additionally, the relatively more polar volatile flavor compounds, such as methyl butanoate, butanoic acid and hexanoic acid, could be less strongly bound by the modified starch.

The interactions between modified starch and water (*x*_1_ × *x*_4_) and between WPI and soursop oil (*x*_2_ × *x*_3_) had positive significant (*p* < 0.05) effects on all the target flavor volatile compounds (Tables 2 and 3). Thus, these results indicated that the interactions between WPI molecules and all of the volatile flavor compounds were either very weak or reversible at low pH [37,38]. Lubbers et al. [37] reported that, in most cases, the chemical interactions between proteins and flavor compounds involved weak hydrophobic and hydrogen bonding. In addition, the soursop volatile flavor compounds are classified as having short to medium carbon chain length. Out of ethyl hexanoate, ethyl octanoate and ethyl nonanoate, only ethyl nonanoate was found to have significant (*p* < 0.05) binding with beta-lactoglobulin [38]. Therefore, the results demonstrated that, within the same chemical class, the affinity of beta-lactoglobulin increased with increasing carbon chain length or hydrophobicity, which suggested hydrophobic interactions.

### The interaction effects of tri-components

For interactions involving three components, the interactions involving modified starch, WPI and soursop flavor oil (*x*_1_ × *x*_2_ × *x*_3_) and the interactions involving modified starch, WPI and water (*x*_1_ × *x*_2_ × *x*_4_) had significant (*p* < 0.05) positive effects on the equilibrium headspace concentrations of methyl butanoate, ethyl butanoate, 1-butanol, butanoic acid and hexanoic acid (Tables 2 and 3). Galazka et al. [39] suggested that high pressure treatment could induce the changes in protein-polysaccharide interactions. The presence of a polysaccharide could have either protected the protein against pressure-induced unfolding or enabled the pressure-denatured beta-lactoglobulin to regain some of its secondary structures [39]. With the lack of hydrophobic binding sites, the flavor compounds would be less bound and could contribute to a higher equilibrium concentration of soursop flavor in the headspace.

On the one hand, the interactions of sole emulsifier systems with flavor oil and water, (*x*_1_ × *x*_3_ × *x*_4_ and *x*_2_ × *x*_3_ × *x*_4_) had shown significant (*p* < 0.05) negative effects on the equilibrium headspace concentration of soursop flavor compounds (Tables 2 and 3). The higher *F*-values (Table 3) illustrated that both modified starch and WPI had higher effectiveness in controlling the equilibrium headspace concentration of soursop flavor compounds when used as sole emulsifiers (*x*_1_ × *x*_3_ × *x*_4_ and *x*_2_ × *x*_3_ × *x*_4_) rather than as mixed biopolymers (*x*_1_ × *x*_2_ × *x*_3_). As shown in Table 3, WPI alone (*x*_2_ × *x*_3_ × *x*_4_) was found to be much more effective in controlling the equilibrium headspace concentrations of methyl butanoate, ethyl butanoate, butanoic acid and hexanoic acid, when compared with the mixed biopolymers system (*x*_1_ × *x*_2_ × *x*_3_). Similarly, modified starch-stabilized emulsion (*x*_1_ × *x*_3_ × *x*_4_) was effective in retarding the flavor release of butanoic acid and hexanoic acid.

Meanwhile, the results indicated that the interactions of modified starch, WPI and flavor oil (*x*_1_ × *x*_1_ × *x*_2_ × *x*_3_) as well as modified starch with flavor and water (*x*_1_ × *x*_1_ × *x*_3_ × *x*_4_) had significant (*p* < 0.05) positive effects on the equilibrium headspace concentration of soursop flavor compounds (Table 2). This result could be due to the excess of modified starch in the system, which caused bridging flocculation and hence destabilizing the emulsion system [4,40]. Additionally, the interactions of WPI with modified starch and flavor oil (*x*_1_ × *x*_2_ × *x*_2_ × *x*_3_) as well as WPI with modified starch and water (*x*_1_ × *x*_2_ × *x*_2_ × *x*_4_) were also shown to have a significant (*p* < 0.05) positive impact on the release of volatile flavors from the concentrated beverage emulsion.

Alternatively, the interactions of WPI, flavor oil and water (*x*_2_ × *x*_2_ × *x*_3_ × *x*_4_) and between modified starch, soursop oil and water (*x*_1_ × *x*_3_ × *x*_3_ × *x*_4_) had significant (*p* < 0.05) negative effects on the equilibrium headspace concentration of soursop flavor compounds (Tables 2 and 3). As shown in Table 3, a single emulsifier system had a higher effectiveness in retarding the flavor release of flavor compounds. Besides that, Jouenne and Crouzet [41] showed the flexibility modification of beta-lactoglobulin between pH 3.0 and 9.0, which had contributed significantly (*p* < 0.05) to an increased retention of flavor compounds, was due to the higher accessibility of hydrophobic binding sites on the beta-lactoglobulin molecule. While Guichard [38] reported two different binding sites for flavor compounds on beta-lactoglobulin molecule.

### Optimization and validation of final reduced models for desirable equilibrium headspace concentration of soursop volatile flavor compounds

**Figure 1:**
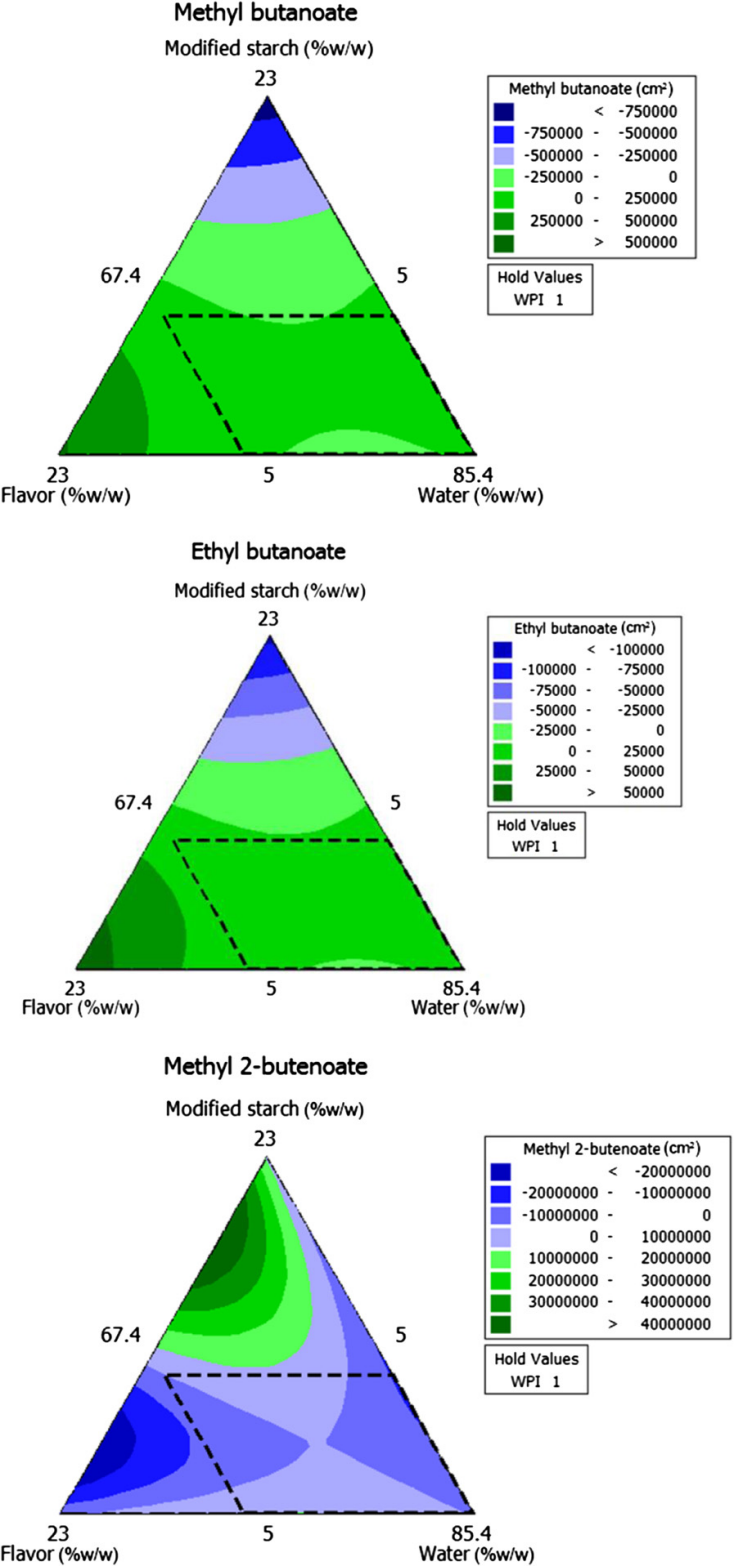
**Mixture contour plots of three soursop flavor compounds.** Diamond-shaped subregion is the constrained experimental region.

**Figure 2:**
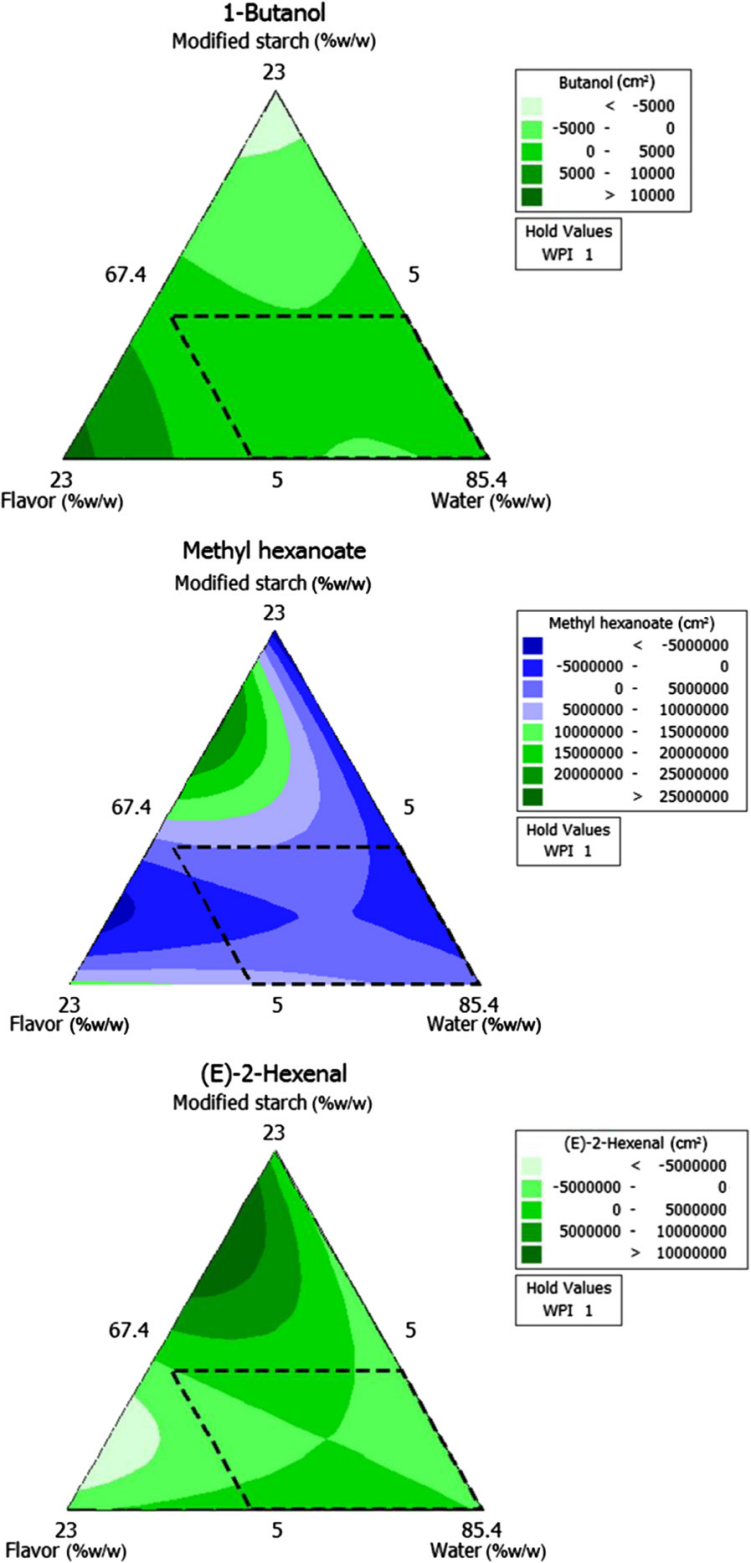
**Mixture contour plots of three soursop flavor compounds.** Diamond-shaped subregion is the constrained experimental region.

**Figure 3:**
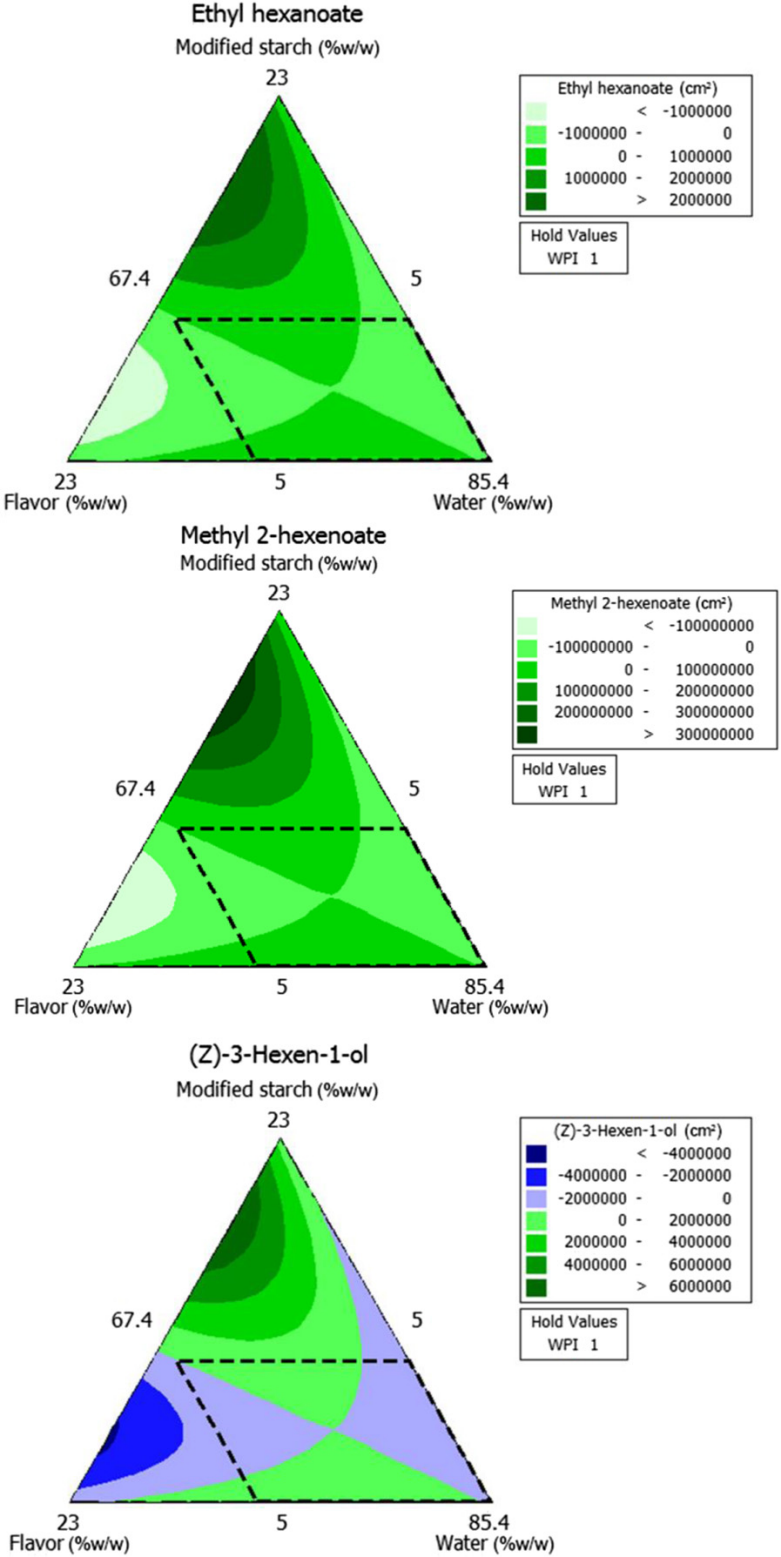
**Mixture contour plots of three soursop flavor compounds.** Diamond-shaped subregion is the constrained experimental region.

**Figure 4:**
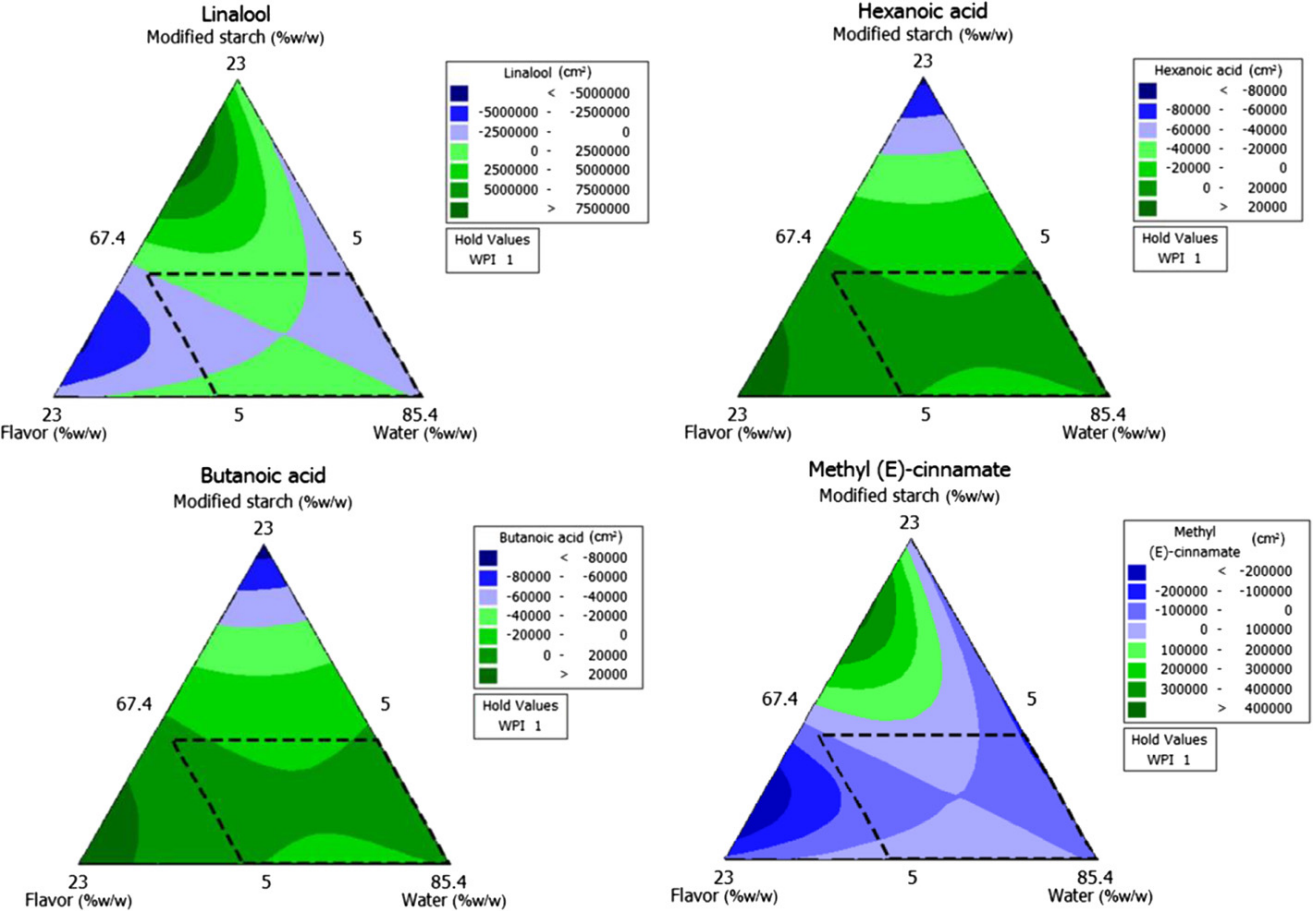
**Mixture contour plots of four soursop flavor compounds.** Diamond-shaped subregion is the constrained experimental region.

The optimization procedure for the equilibrium headspace concentration is determined by the observation of the lowest possible peak area for each of the target volatile flavor compounds. The contour plots were drawn by using the center points of each of the dependent interval (Figures 1,2,3 and 4). A numerical optimization was also performed for the simultaneous multiple optimization of the response variables resulting in the desirable equilibrium headspace concentration of soursop volatile flavor compounds. The multiple optimization results showed that the minimum overall release of soursop volatile flavor compounds could be achieved when the emulsion was formulated using 8.56% (w/w) modified starch, 1.13% (w/w) WPI, 10.27% (w/w) soursop flavor oil and 76.45% (w/w) water. The peak areas estimated for methyl butanoate, ethyl butanoate, methyl 2-butenoate, 1-butanol, methyl hexanoate, (*E*)-2-hexenal, ethyl hexanoate, methyl 2-hexenoate, (*Z*)-3-hexen-1-ol, linalool, butanoic acid, hexanoic acid and methyl (*E*)-cinnamate were 3.18 × 10^4^, 552.01, 4.09 × 10^4^, 503.03, 6.71 × 10^4^, 7433.83, 254.97, 2.74 × 10^5^, 2616.52, 2294.39, 1239.17, 1558.36 and 198.50, respectively.

Previous studies [26,38,42] have also demonstrated that interfacial interactions between hydrocolloids/emulsifiers and aroma compounds could limit the transfer of hydrophobic compounds from oil to water. However, formulations with high excess of modified starch were susceptible to destabilization via depletion flocculation [4,40]. To verify the adequacy of the final regression models, experimental values were statistically compared with the predicted values by using a two-sample T-test. No significant difference (*p* > 0.05) was reported between the actual and the predicted values.

## Conclusions

The present study showed that the equilibrium headspace concentration of soursop volatile flavor compounds were significantly (*p* < 0.05) influenced by the composition of soursop beverage emulsion. Interactions between modified starch and protein were found to have antagonistic effects on the flavor release from the emulsion matrix. This might be explained by the fact that the film formed using the combination of both modified starch and WPI were capable of providing a barrier to inhibit the release of volatile flavor compounds from the oil to the aqueous phase. The negative effect of modified starch on the overall flavor release may also be attributed to the physical entrapment of flavor compound molecules through its viscosity enhancement effect, which impeded the flavor release of volatile compounds across the emulsion matrix. Yet, the present study also revealed significant differences between the use of individual emulsifiers and combination of both emulsifiers. The *F*-values for systems containing single emulsifier were higher than those containing a mixture of both modified starch and WPI. The pressure-induced protein-polysaccharide interactions could have contributed to the lower hydrophobic binding sites on the beta-lactoglobulin molecules, which explained the positive effects of interaction between WPI and flavor oil (*x*_2_ × *x*_3_) for all the flavor compounds studied. In contrast, both modified starch and WPI showed to be a much more effective barrier in inhibiting the release of all the soursop flavor compounds studied when used as individual emulsifier. The current study revealed that the polar volatile compound such as 1-butanol showed a relatively higher affinity for water phase than the oil phase. Thus, it was less bound by both modified starch and WPI. This study showed the importance of understanding the interaction effects between emulsion components for developing an optimum beverage emulsion with desirable flavor release profile. In addition, it also showed that the general equilibrium headspace concentration of soursop flavor compounds from the emulsion beverage could be modified by the proportion of the main emulsion components.

## Experimentals

### Chemicals and materials

The octenyl succinate (OSA) modified waxy maize starch (Purity Gum 1773) was a gift from National Starch and Chemical (Bridgewater, NJ, USA). Whey protein isolate (Provon A190) was provided by Glanbia Nutritionals (Monroe, WI, USA). The soursop flavor oil was provided by Flavor Inn Corporation (Selangor, Malaysia). The palm olein was purchased from a local retailer. Citric acid (Sigma-Aldrich, St. Louis, MO, USA) was used to adjust pH of emulsion. Sodium benzoate (Sigma-Aldrich, St. Louis, MO, USA) and potassium sorbate (Acros Organics, NJ, USA) were used as preservatives in the beverage emulsion system. Sodium chloride (NaCl) was purchased from Merck (Darmstadt, Germany). Deionized water was used to prepare the beverage emulsions. The solid-phase assembly holder, 75 μm carboxen/polydimethylsiloxane (CAR/PDMS) fiber, butyl rubber septa, 20-mL glass vials and aluminum vial crimp seals were supplied by Supelco Inc. (Bellefonte, PA, USA).

### Preparation of soursop beverage emulsions

In the present study, 17 soursop beverage emulsions composed of modified starch (5-12% w/w), WPI (0-2% w/w), soursop oil (5-15% w/w), deionized water (67.4-86.4% w/w), vegetable oil (3% w/w), sodium benzoate (0.1% w/w), potassium sorbate (0.1% w/w) and citric acid (0.4% w/w) were prepared for the optimization procedure based on a four-component, constrained extreme vertices mixture design (Table 1). To prepare the aqueous phase, sodium benzoate, potassium sorbate and citric acid were sequentially dispersed in deionized water that was kept stirred using a magnetic stirrer. Subsequently, WPI and modified starch were also dispersed in succession in the deionized water. The mixture was then left at room temperature while being stirred for 2 hours to facilitate hydration. While mixing the water phase using a high speed Waring blender (32BL80, New Hartford, USA), the soursop flavor oil was gradually added into the aqueous phase to form an initial coarse emulsion [43]. Fine emulsification (*e.g.*, small average droplet size of < 1 μm with a narrow particle size distribution) was achieved by subjecting initial coarse emulsion to pre-homogenization using a high-shear homogenizer (Silverson L4R, Buckinghamshire, UK) for 1 min at 6,000 rpm and then passing it through a high-pressure homogenizer (APV, Crawley, UK) for 2 cycles at 200 bar.

### Equilibrium headspace analysis using HS-SPME

For the HS-SPME analysis, 5 g of diluted soursop beverage emulsion (5% w/w) was transferred into a 20-mL vial containing NaCl (30% w/w) and a micro-magnetic stirring bar. Subsequently, the vial was sealed with a Teflon-lined septum and immersed in a water bath at a fixed temperature, 25°C. The sample was continuously stirred for 15 min at 25°C prior to sampling using CAR/PDMS fiber, which was manually exposed to the sample headspace for 10 min to reach equilibrium. The sample was continuously agitated with a magnetic stirring bar during the extraction process to allow a more certain establishment of equilibrium conditions. Subsequently, the fiber was withdrawn into the needle then introduced into the gas chromatography injection port and held there for 5 min to completely desorb the volatile flavor compounds [44].

### Gas chromatography-flame ionization detector (GC-FID) conditions

The quantitative equilibrium headspace analysis of soursop volatile flavor compounds was performed using an Agilent 6890N GC (Palo Alto, CA, USA) equipped with a flame ionization detector (FID) and a DB-Wax capillary column (i.d. = 0.25 mm, length = 30 m, film thickness = 0.25 μm) (J & W Scientific, Folsom, CA, USA). The GC injection port was equipped with a 0.75 mm i.d. liner (Supelco, Bellefonte, PA, USA) to minimize peak broadening. For the equilibrium headspace analysis of soursop beverage emulsion, the injection was performed in a splitless mode for 5 min at 250°C. The flow rate of the carrier gas, helium, was set at a constant flow rate of 1.4 mL/min. The oven temperature was programmed at 40°C isothermally for 3 min, then ramped to 120°C at 2°C/min and subsequently raised up to 250°C at 20°C/min and held for 5 min at a final temperature of 250°C. Injector and detector temperatures were 250°C and 270°C, respectively [33,44].

### Experimental design and data analysis

A four-component with constrained extreme vertices design was used to formulate the soursop beverage emulsions. In this study, a mixture design comprising 17 soursop beverage emulsion formulations using modified starch (*x*_*1*_), WPI (*x*_*2*_), soursop flavor oil (*x*_*3*_), deionized water (*x*_*4*_), vegetable oil, sodium benzoate, potassium sorbate and citric acid were constructed to study the effect of different concentrations of modified starch, WPI, soursop flavor oil and deionized water on the equilibrium headspace concentration of volatile flavor compounds of soursop. In mixture design, all of the components and their levels are not independent of each other, as the sum of the proportions of the mixture components is always 1 [45]. The minimum and maximum levels of each mixture component were modified starch (5-12% w/w), WPI (0-2% w/w), soursop oil (5-15% w/w) and deionized water (86.4-67.4% w/w). The experiments were replicated and randomized in order to minimize the effect of unexplained variability in the actual responses due to extraneous factors. Analysis of variance (ANOVA) and regression surface analysis were conducted to determine the statistical significance of the model terms and to fit a regression relationship relating the experimental data to dependent variables resulting in desirable goals [43]. The experimental design and data analysis were performed using Minitab release 14.20 statistical package (Minitab Inc., State College, PA, USA).

### Optimization and validation procedures

A numerical optimization was carried out using response optimizer function in the Minitab software for simultaneous optimization and to determine the exact optimum levels of these variables (*x*_*1*_, *x*_*2*_, *x*_*3*_ and *x*_*4*_) leading to the desired equilibrium headspace concentration of soursop flavor compounds. In this study, a low release of volatile flavor compounds from the concentrated beverage emulsion would be considered to be an ideal system. The adequacy of the regression equations was checked by comparing the experimental data with predicted values obtained from the equations [43].

## Competing interests

The authors declared that they have no competing interests.

## Authors’ contributions

KWC carried out all the experiments, data analysis and interpretation of data. KWC also prepared the draft manuscript, while CPT, HM and MB have critically reviewed the content of this manuscript. WYJK, NSAH and AO have made intellectual contributions and given the final approval for the manuscript to be published.
